# Supplementary material for: OverFlap PCR: A reliable approach for generating plasmid DNA libraries containing random sequences without a template bias
Source: PLoS One. 2022 Aug 8;17(8):e0262968. doi: 10.1371/journal.pone.0262968 (PMC9359533; doi:10.1371/journal.pone.0262968)
Supplement: S1 Fig — Creating the p426GPD-aFactor-aMSH plasmid involved supplementing the standard p426GPD vector with a Saccharomyces cerevisiae yeast α-factor secretion signal and the α-melanocyte stimulating hormone fusion protein-coding sequence. BamHI and EcoRI restriction sites were used for this purpose. This study used an XhoI restriction site for plasmid linearization before OverFlap PCR. (PDF) [file pone.0262968.s001.pdf]

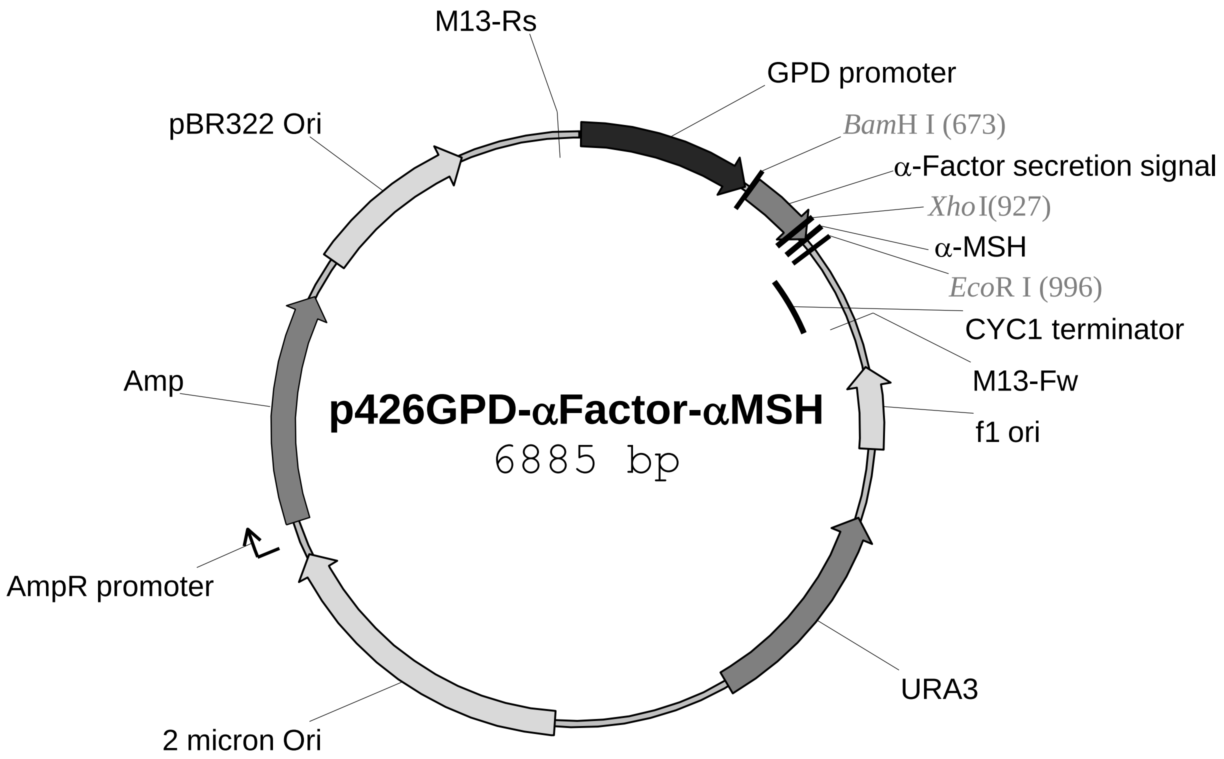

Supplementary Figure 1. The feature map of created p426GPD-αFactor-αMSH plasmid that was used in this study for generation of plasmid library for random peptide expression. Its creation involved supplementation of standard p426GPD vector with yeast *Saccharomyces cerevisiae* α–Factor secretion signal and α-Melanocyte Stimulating Hormone fusion protein coding sequence. *Bam*HI and *Eco*RI restriction sites were used for this purpose. *Xho*I restriction site was used during this study for plasmid linearization prior OverFlap PCR.
