# Supplementary material for: OverFlap PCR: A reliable approach for generating plasmid DNA libraries containing random sequences without a template bias
Source: PLoS One. 2022 Aug 8;17(8):e0262968. doi: 10.1371/journal.pone.0262968 (PMC9359533; doi:10.1371/journal.pone.0262968)
Supplement: S2 Fig — The red, black, grey, and dotted lines represent the randomized region, template DNA, synthesized chain, and DNA synthesis, respectively. (PDF) [file pone.0262968.s002.pdf]

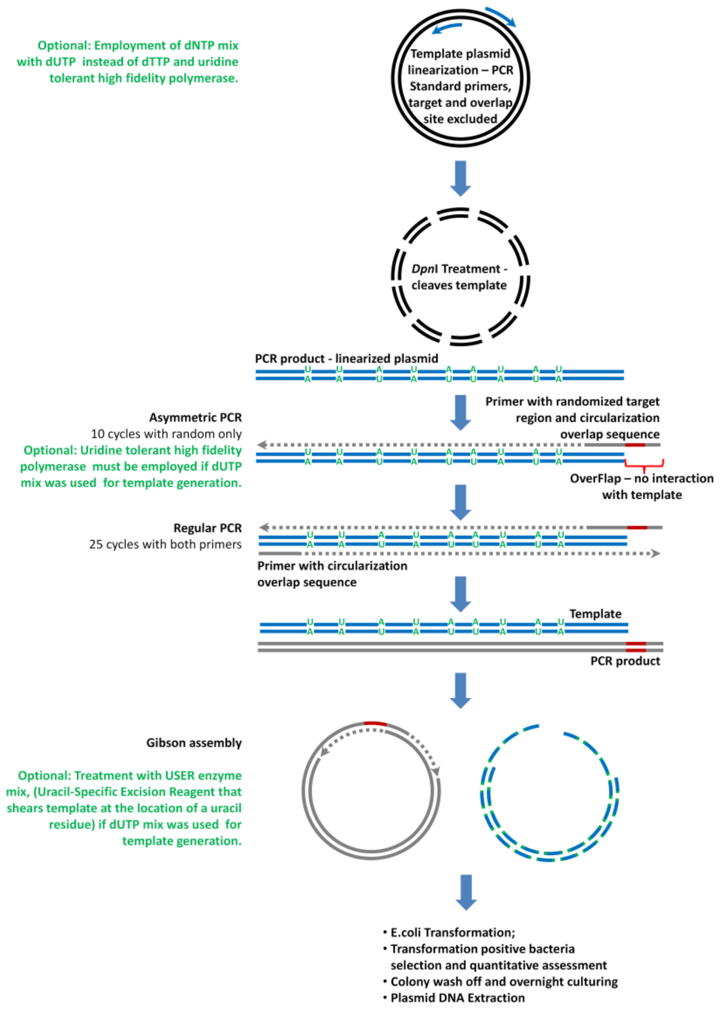

The principal scheme of proposed future OverFlap whole plasmid amplification based randomization of selected plasmid DNA region. Randomized region is represented by red line; the template DNA is represented by black line; the synthesized chain is represented by grey line; Dotted line represents DNA synthesis.

The linearization of template should be performed employing PCR based whole plasmid amplification. Here, as an option, the employment of reaction mix with dUTP instead of dTTP and uridine tolerant high fidelity polymerase is applicable. The primers should have perfect complementarity to template and should be designed to exclude both randomization target region and circularization overlap region from linearized randomization template. Acquired product should be treated with *Dpn*I and purified preferably with some size selection methodology (agarose gel excision or magnetic beads) to exclude remaining fragments of template, any nonspecific PCR products and primer dimers. Acquired product should then be used as template for randomization through Asymmetric OverFlap Whole Plasmid Amplification, where first 10 or more PCR cycles are

performed employing only randomization primer that also contains overlap sequence and additional 25 cycles are performed employing both primers. If dUTP was used then at this stage acquired products should be treated with USER enzyme mix and purified if not then just purified. Thus acquired randomized liner DNA should be circularized employing Gibson assembly and transformed through electroporation in competent *E.coli* cells of suitable strain. Cells should then be seeded on petri dish to assess the number of transformation positive colonies and thus the theoretical maximal diversity of created library. After that colonies should be washed off and inoculated in liquid media for additional propagation prior plasmid extraction. The assessment of randomization success and diversity of acquired library should be performed employing suitable sequencing technology. The randomization procedure should be repeated each time new plasmid library stock is needed. However if frozen stocks are created then it should be stored in aliquots of sufficiently large bacterial cell number to maintain acquired diversity of random plasmid library and whole volume of single the aliquot should be used for inoculation.
