# Supplementary material for: OverFlap PCR: A reliable approach for generating plasmid DNA libraries containing random sequences without a template bias
Source: PLoS One. 2022 Aug 8;17(8):e0262968. doi: 10.1371/journal.pone.0262968 (PMC9359533; doi:10.1371/journal.pone.0262968)
Supplement: S1 Raw images — (PDF) [file pone.0262968.s004.pdf]

Figure 3a Original

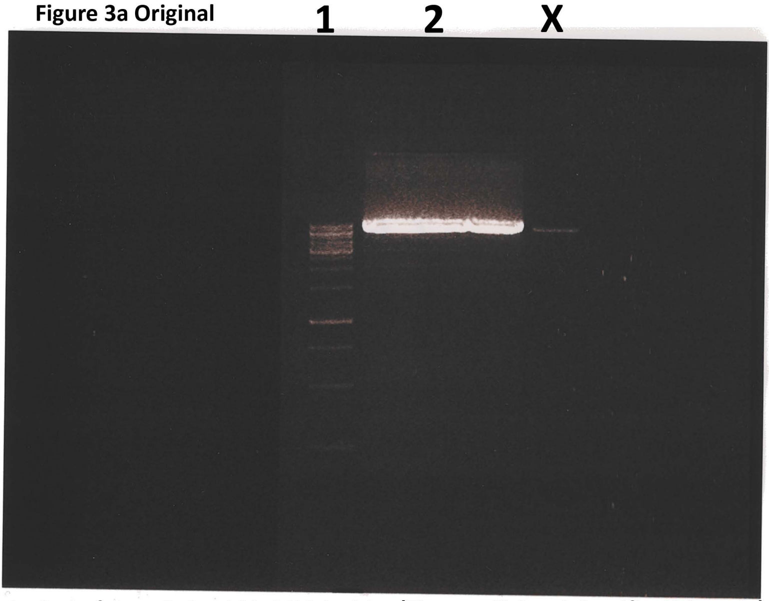

1 - 2 ul of GeneRuler 1 kb DNA Ladder (Thermo Fisher Scientific, Lithuania)
2 - 5 ul sample of products prior DpnI and USER enzyme mix treatment
Image was captured by monochromatic digital camera - thermal printer system

Figure 3b Original

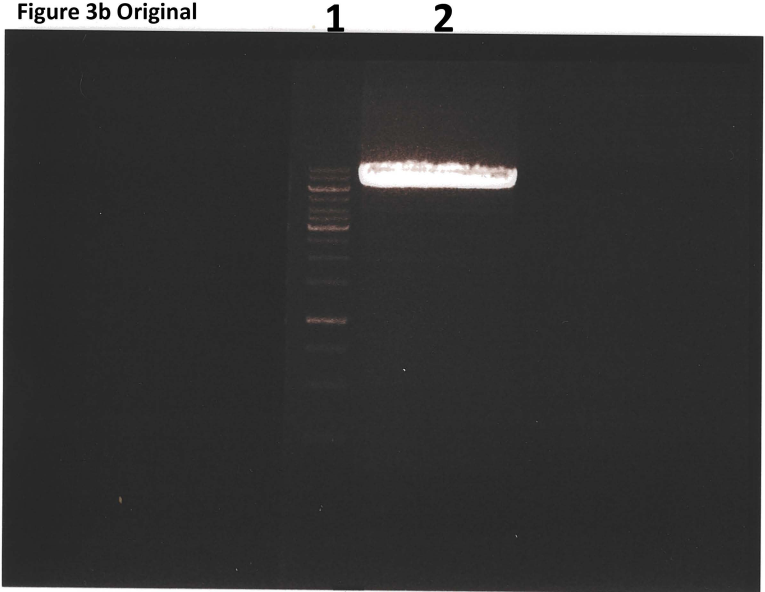

1 - 2 ul of GeneRuler 1 kb DNA Ladder (Thermo Fisher Scientific, Lithuania)
2 - 5 ul sample of products after DpnI and USER enzyme mix treatment
Image was captured by monochromatic digital camera - thermal printer system
