# Supplementary material for: OverFlap PCR: A reliable approach for generating plasmid DNA libraries containing random sequences without a template bias
Source: PLoS One. 2022 Aug 8;17(8):e0262968. doi: 10.1371/journal.pone.0262968 (PMC9359533; doi:10.1371/journal.pone.0262968)
Supplement: Attachment — Submitted filename: Responses to Reviewers v2.doc [file pone.0262968.s006.doc]

# Responses to Reviewers Comments

**Responses to Reviewer #1 Comments:**

*Comment:	1. The authors have created a diverse library of secreted peptides. Won’t secretion sever the link between genotype and phenotype? How would they identify clones with biological function?*

**Response:**	We are grateful for the Reviewer’s insight and concern. We agree that maintaining the link between genotype and phenotype is a major issue in some applications of random peptide expression libraries, especially in those that involve secretion. However application of created random peptide library expression plasmids is not the subject of this article. We provided marginal insight into our future plans only to explain why the assessment of randomization process efficiency was carried out on the encoded amino acid sequence level and not on nucleic acid sequence level, but our research activities are being continued and if the reviewer would be so kind to reveal him/her self by providing the e-mail address, we will be happy to send the link to the preprint of our next article as soon as it will be finished and made available to public.

*Comment:	2. How is cumulative occurrence probability (COP), now calledk CPO, computed?*

**Response:**	We thank Reviewer for the comment and we understand the confusion that this change in terminology might have caused. However we have already explained the reasons behind these changes in our response to one of reviewer’s previous comments (Comment No. 7), therefore we decided to abstain from provision of essentially identical information, but for the convenience of review process we are including both previous comment and its response as an inline element of our current response:

“*Reviewers* *previous* *Comment: 7. The calculation of cumulative occurrence probability (COP) ought to be spelled out so that all readers (including this reviewer) can understand it.”*

“Our previous Response**:** We appreciate the comment of the reviewer. Not being the native English speakers, we were are aware that there might be some grammar issues in our manuscript, therefore we double checked the spelling of “cumulative occurrence probability (COP)” everywhere within the manuscript, but unfortunately were unable to identify any spelling related issue. Thus we presumed that reviewer was either referring to incorrectness in choice of our terminology or to insufficiency of explanations on this subject. To comply with the first possibility we decided to replace selected term “cumulative occurrence probability (COP)” with the alternative: “Cumulative probability of occurrence (CPO)” and we hope that reviewer will find this change as acceptable. To comply with the second possibility, we added additional explanations that describe assumptions and calculations behind CPO and these changes will satisfy the reviewer.”

**Responses to Reviewer #2 Comments:**

*Comment:	Authors have addressed my concerns and made other changes that feel improved the manuscript. The changes the authors made to the figures improved clarity of the technique and the re-write in explaining the rational behind CPO was helpful. I feel the manuscript is technically and grammatically sound and of value.*

**Response:**	Once again we are grateful for the reviewer’s kind words and we are happy that improvements of the manuscript were found suitable for publication .
